# Ovarian dysfunction in women with Turner syndrome

**DOI:** 10.3389/fendo.2023.1160258

**Published:** 2023-03-23

**Authors:** Maki Fukami

**Affiliations:** Department of Molecular Endocrinology, National Research Institute for Child Health and Development, Tokyo, Japan

**Keywords:** BMP15, oocyte apoptosis, premature ovarian insufficiency, pairing failure, streak gonad

## Abstract

Ovarian dysfunction is one of the most common features of women with Turner syndrome. In these women, oocyte apoptosis is markedly accelerated from the early stage of fetal life. Reduction in the number of germ cells disturbs primordial follicle development and thereby leads to the formation of streak gonads. There are three possible causes of accelerated germ cell loss in 45,X ovaries. First, chromosomal pairing failure due to X chromosomal aneuploidy is believed to induce meiotic arrest. Indeed, it has been suggested that the dosage of the X chromosome is more critical for the survival of the oocytes than for other cells in the ovary. Second, impaired coupling between oocytes and granulosa cells may also contribute to germ cell apoptosis. Previous studies have shown that 45,X ovaries may tend to lose tight junctions which are essential for intercellular interactions. Lastly, ovarian dysfunction in women with Turner syndrome is partly attributable to the reduced dosage of several genes on the X chromosome. Specifically, *BMP15*, *PGRMC1*, and some other genes on the X chromosome have been implicated in ovarian function. Further studies on the mechanisms of ovarian dysfunction are necessary to improve the reproductive outcomes of women with Turner syndrome.

## 1 Introduction

The most common features of women with Turner syndrome are ovarian dysfunction and short stature (1). These women typically present with primary amenorrhea, and only 10–15% of them manifest spontaneous menarche (2, 3). Since spontaneous pregnancy and delivery have been reported in multiple cases (4), there seem to be large inter-individual variations in the severity of ovarian dysfunction. In particular, the ovarian phenotype tends to be milder in women with 45,X/46,XX mosaicism than in those with a non-mosaic 45,X karyotype (3).

In adulthood, most women with Turner syndrome present with premature ovarian insufficiency (POI), that is, early menopause due to ovarian dysfunction (5). POI is a relatively common disorder in the general population which affects approximately 1% of women under 40 years of age (5). Various genetic and environmental factors have been implicated in the development of POI (5, 6). Of these, numerical or structural abnormalities of the X chromosome, including Turner syndrome and X chromosomal trisomy, represent the major genetic causes (7). This mini-review introduces the typical ovarian features of women with Turner syndrome and the possible mechanisms of the features.

## 2 Ovarian features of women with Turner syndrome

### 2.1 Reduced germ cell number

The key ovarian finding in Turner syndrome is the reduced number of oocytes (8, 9). Previous studies have suggested that in 45,X ovaries, the initial differentiation and migration of germ cells remain unaffected; however, prominent germ cell loss occurs during embryonic stages (9, 10). Of note, progressive germ cell loss during the life course is a physiological phenomenon in all women. In a healthy woman, the germ cell number usually reaches a maximum value of ~6 ×10^6^ at the mid-gestation fetal stage and constantly decreases thereafter (11, 12). The estimated number of germ cells is ~1–2 ×10^6^ at birth, ~3 ×10^5^ at the onset of puberty, and < 1,000 at the time of menopause (11). This decline in the oocyte number is independent of ovulation and was linked to apoptosis (11, 13). In normal ovaries, apoptosis of a small percentage of germ cells is observed throughout the meiotic prophase as well as during folliculogenesis (12). In women with Turner syndrome, oocyte apoptosis is markedly accelerated from the early stage of fetal life. Modi et al. reported that approximately 70% of germ cells were apoptotic in the ovaries of 45,X fetuses at 20 weeks of gestation, while only 3%–5% of germ cells were apoptotic in the ovaries of age-matched 46,XX fetuses (12). Lundgaard Riis et al. also confirmed an increased number of apoptotic cells in 45,X ovaries at 12–20 weeks of gestation (9). Thus, Turner syndrome appears to be characterized by premature exhaustion of the germ cell reservoir due to accelerated apoptosis (11). Moreover, the prominent oocyte loss may not solely be attributed to increased apoptosis. Modi et al. proposed the presence of other mechanisms for the physiological germ cell loss in 46,XX fetuses, based on the observation that the number of apoptotic cells observed in the ovaries is smaller than the estimated number of cells lost during the fetal period (12).

### 2.2 Impaired primordial follicle formation

During normal ovarian development, oocytes are arrested at the diplotene stage of the first meiotic division and are surrounded by single-layer granulosa cells to form primordial follicles (9, 14). Lundgaard Riis et al. reported that the number of primordial follicles in 45,X ovaries at 16–24 weeks of gestation was significantly lower than that in age-matched 46,XX ovaries, whereas the number in the mosaic 45,X/46,XX ovaries was moderately reduced (9). In addition, postnatal ovarian biopsies by Mamsen et al. showed that follicles were present only in about 60% of girls with Turner syndrome, mostly in those with mosaic karyotypes (15).

Defective folliculogenesis in 45,X ovaries likely reflects the decreased number of germ cells, as follicular development is known to require bidirectional signaling between the oocyte and the surrounding granulosa cells (16). Mamsen et al. reported that most ovaries of girls with Turner syndrome had abnormal follicle morphology, such as vacuolated oocytes and incomplete layers of granulosa cells surrounding the oocyte (15). Furthermore, Reynaud et al. reported impaired transformation of pre-granulosa cells to granulosa cells in 45,X ovaries at an early fetal stage (17). In addition, follicle fluid samples obtained from the small follicles of girls with Turner syndrome contained relatively low levels of estradiol and testosterone and high levels of anti-Müllerian hormone, indicating an impaired function of ovarian somatic cells (15).

### 2.3 Streak gonads and hypergonadotropic hypogonadism

Most women with Turner syndrome have streak gonads or immature ovaries and manifest hypergonadotropic hypogonadism (18). Defective gonadal development in these women reflects the lack of germ cells and defective primordial follicle formation during the fetal period. Indeed, it is known that the presence of germ cells is essential for ovarian development, and oocyte loss before the pachytene stage leads to ovarian dysgenesis (19).

In women with Turner syndrome, hypergonadotropic hypogonadism is usually apparent from childhood. Increased blood levels of gonadotropins can be ascribed to the defective negative feedback of estrogens on the hypothalamus/pituitary function. Interestingly, central precocious puberty has been documented in a few patients with Turner syndrome and the mosaic 45,X/46,XX karyotype. The mechanism of precocious puberty in these girls remains unclear, but may be associated with an aberrant FSH surge preceding the development of ovarian dysfunction (20).

## 3 Possible causes of the ovarian phenotype of Turner syndrome

### 3.1 Chromosomal pairing failure during meiosis

One of the possible causes of germ cell loss in Turner syndrome is chromosomal pairing failure during meiosis (21). Human meiosis involves crossing-over between two homologous chromosomes (22). Thus, aneuploidy or large structural alterations of a chromosome result in pairing failure and resultant meiotic arrest (23, 24). Consistent with this, a systematic review demonstrated that non-mosaic sex chromosomal abnormalities leading to severe pairing failure were frequently associated with primary amenorrhea and/or streak gonads (**Table 1**) (1). Likewise, X-chromosomal trisomy (47,XXX) and balanced X-autosome translocations have been reported as risk factors of POI (2). In this context, heterozygous deletions encompassing the entire short arm pseudoautosomal region, the platform of sex chromosomal pairing during male meiosis (23, 24), were shown to cause spermatogenic arrest in men (25, 26). Notably, however, phenotypic consequences of sex chromosomal pairing failure in oocytes may differ among species. X-monosomic oocytes in mice can escape elimination through non-homologous self-synapsis of the single X chromosome (27).

**Table 1:** Review of clinical features and genetic factors of women with 45,X monosomy and non-mosaic X chromosomal structural abnormalities.

Karyotype	Number of cases	Clinical features		Genetic factors		
		Primary amenorrhea/streak gonad	Secondary amenorrhea/abnormal menses	Pairing failure of sex chromosomes	Copy-number of Xp genes	Copy-number of Xq genes
45,X	103	88%	12%	Severe	1	1
46,X,i(Xq)	35	91%	9%	Severe	1	3
46,X,idic(Xq)	10	80%	20%	Severe	3	1 or 3
46,X,idic(Xp)	11	73%	27%	Severe	1 or 3	3
46,X,del(X)(p22.3)	15	0%	0%	Slight	1 or 2	2
46,X,del(X)(p22.2-p21)	8	13%	25%	Mild	1 or 2	2
46,X,del(X)(p11)	40	50%	45%	Moderate	1 or 2	2
46,X,del(X)(q13-q21)	32	69%	31%	Moderate-severe	2	1 or 2
46,X,del(X)(q22-q25)	16	31%	56%	Moderate	2	1 or 2
46,X,del(X)(q26-q28)	12	8%	67%	Mild	2	1 or 2
46,X,del(Xp)(interstitial)	4	25%	25%	Mild	1 or 2	2
46,X,del(Xq)(interstitial)	8	0%	63%	Mild	2	1 or 2
46,X,t(X;autosome)	74	28%	24%	Mild-moderate	2	2
46,X,inv(X)	20	5%	20%	Mild	2	2
46,X,Yp-	2	100%	0%	Severe	1	1
47,XXX	46	4%	37%	Mild	3	3

This table is based on the data reported by Ogata and Matsuo (1).

Peek et al. performed cytogenetic and morphological analyses of ovarian tissues obtained from 10 girls with Turner syndrome (28). The authors detected small ovarian follicles only in five girls with mosaic 45,X/46,XX or 45,X/46,XX/47,XXX karyotypes, but not in the other five girls with a non-mosaic 45,X karyotype. Interestingly, in the small follicles of the mosaic ovaries, most oocytes were euploid, whereas the granulosa cells were largely monosomic and the stromal cells showed high levels of mosaicism. This suggests that the dosage of the X chromosome is more critical for the survival of the oocytes than for other cells in the ovary. In addition, because ovarian morphology was normally preserved in a case where 90% of stromal cells were 45,X, it appears that sex chromosomal aneuploidy in somatic cells exerts only minimal effects on ovarian morphology. Notably, ovarian function was less severely affected in Turner syndrome women with 45,X/47,XXX mosaicism, with or without the presence of a 46,XX cell line, than in those with non-mosaic 45,X karyotype (29, 30). Actually, most women with 45,X/47,XXX karyotype exhibit spontaneous menarche and early menopause (29, 30). It remains to be clarified how the presence of a 47,XXX cell line can decelerate age-dependent germ cell loss in 45,X ovaries.

### 3.2 Defective intercellular interaction in the ovaries

Chromosomal pairing failure may not be the sole cause of accelerated germ cell apoptosis in Turner syndrome. Indeed, in 45,X ovaries, impaired oogenesis was observed at a very early stage of meiotic prophase, even before chromosomal pairing (12). Modi et al. proposed that impaired coupling between the oocyte and granulosa cells leads to germ cell apoptosis in Turner syndrome (12). This assumption is based on the findings that (i) coupling of granulosa cells among themselves and with oocytes *via* the gap junctions and tight junctions are essential for the maintenance of oocytes in the fetal ovary, and (ii) multiple adult women with a 45,X karyotype were found to lack tight junctions in the endometrium. However, it remains uncertain as to whether the gap junctions and tight junctions of granulosa cells are actually impaired in fetuses with Turner syndrome. In addition, hitherto unknown mechanisms may also contribute to the accelerated germ cell loss in Turner syndrome.

### 3.3 Dosage effects of X chromosomal genes

**Figure 1:**
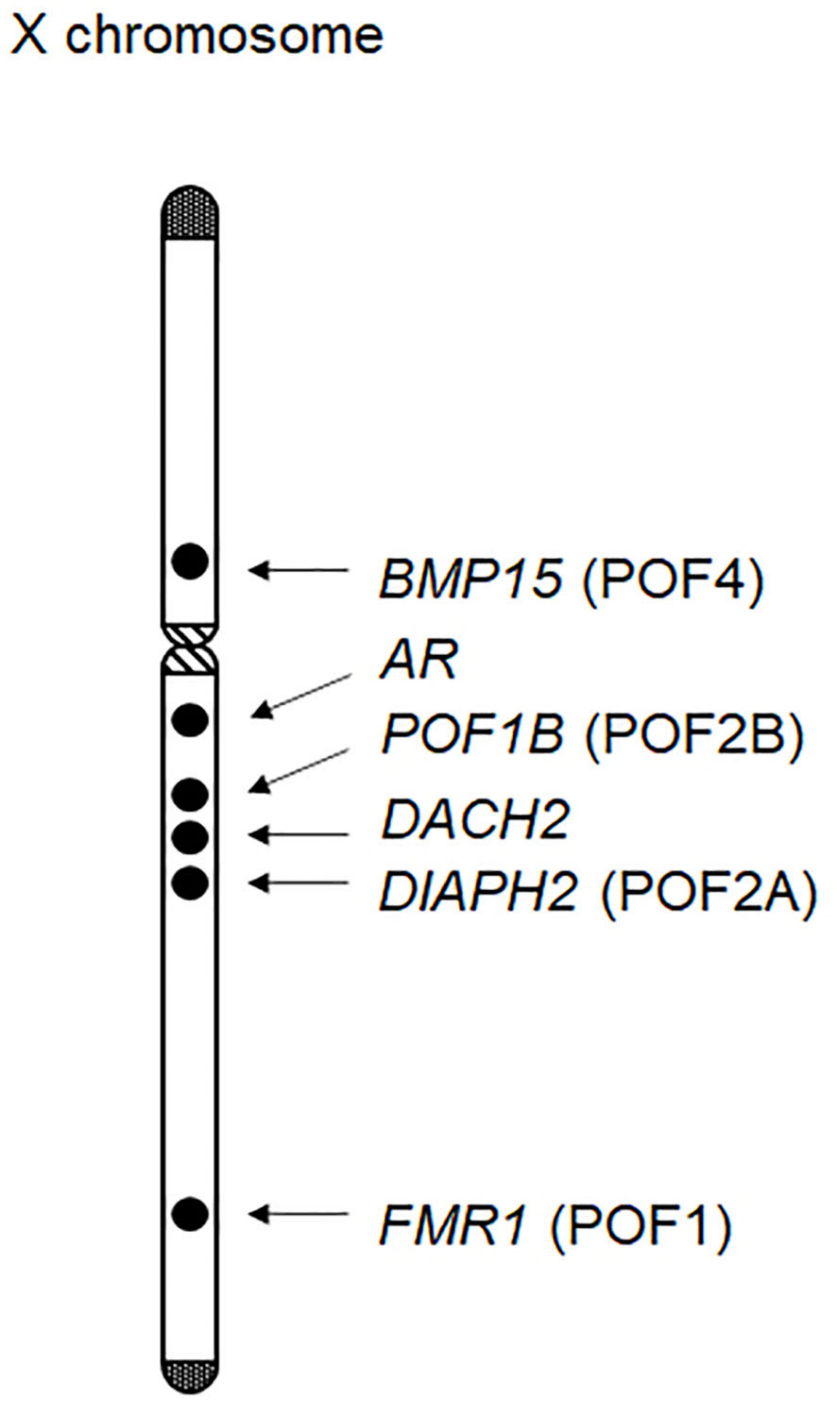
X chromosomal genes possibly associated with ovarian function. At least six genes have been implicated in ovarian development or function. Of these, *BMP15*, *POF1B*, *DIAPH2*, and *FMR1* have been designated as premature ovarian failure (POF) loci.

Ovarian dysfunction in women with Turner syndrome is partly attributable to the reduced dosage of some genes on the X chromosome. The human X chromosome contains more than 1,000 genes (the UCSC Genome Browser; https://genome.ucsc.edu/). Haploinsufficiency of some X chromosomal genes, particularly that of X chromosome inactivation-escape genes, potentially underlies the Turner syndrome phenotype (31). Indeed, *SHOX* at Xp22.33 is known as the main causative gene for short stature in women with Turner syndrome (32). Moreover, several X chromosomal genes were reported to be involved in the development or function of ovaries (**Figure 1**). Castronovo et al. proposed that the dosage effect of X chromosomal genes is the major factor of the ovarian dysfunction in Turner syndrome, based on the observation that spontaneous menarche was significantly more common in patients with a high level of 45,X/46,XX mosaicism than in non-mosaic patients (33). Genotype-phenotype correlation studies suggested that genes on Xp are particularly important for ovarian function (**Table 1**) (1, 34).

*BMP15* at Xp11.2 is known to be involved in follicular development (35). *BMP15* encodes a member of the TGF-β superfamily and is expressed in the oocytes throughout folliculogenesis. *Bmp15* knockout female mice exhibit decreased ovulation rates and a reduced number of litters per lifetime (36). In 2004, Di Pasquale et al. identified a rare heterozygous variant of *BMP15* in patients with ovarian dysgenesis (37). The p.Y235C variant was shared by two affected sisters, and exerted dominant-negative effects on wildtype BMP15 in cultured cells. Subsequently, multiple rare variants of *BMP15* were detected in patients with POI (38). For example, Rossetti et al. performed *BMP15* mutation screening for 300 women with POI and identified three probable pathogenic variants (p.L148P, p.R138H, and p.R68W) in five individuals (39). *In vitro* assays showed that these *BMP15* variants impaired mature protein production. Furthermore, two heterozygous frameshift variants of *BMP15* were identified in patients with POI (40). These results indicate that appropriate amounts of the BMP15 protein are essential for ovarian follicle development. Considering that *BMP15* is deleted in most women with Turner syndrome, haploinsufficiency of this gene may play a significant role in the etiology of POI in this condition.

*PGRMC1* at Xq22-q24 is expressed in various tissues, including the ovary, and has been implicated in progesterone signaling in the reproductive system. *PGRMC1* encodes a putative progesterone-binding membrane receptor (2) and likely mediates the anti-apoptotic effects of progesterone on granulosa cells. Mansouri et al. proposed a link between POI and reduced *PGRMC1* expression in a mother-daughter pair with an X;autosome translocation (41). Moreover, the p.H165R variant of *PGRMC1*, which was predicted to attenuate protein function, was identified in a patient with POI (41). Thus, *PGRMC1* haploinsufficiency may also contribute to the development of POI in women with Turner syndrome.

Several other X chromosomal genes may also be associated with POI of Turner syndrome. For example, previous studies have identified rare sequence variants of *POF1B* at Xq21.1-21.2 and those of *DACH2* at Xq21.2 in some patients with POI (42). Similarly, *DIAPH2* at Xq21.33 was reported as a candidate gene for POI, based on the observation that two women with balanced translocation close to this gene showed ovarian dysfunction (43). In addition, triplet repeat expansion in *FMR1* is known to cause POI (44), although the association between *FMR1* haploinsufficiency and ovarian dysfunction remains unknown. Notably, *FMR1*, *DIAPH2*, *POD1B*, and *BMP15* were previously designated as premature ovarian failure (POF) loci, namely, POF1, POF2A, POF2B, and POF4, respectively (**Figure 1**). In addition, rare variants in the androgen receptor gene (*AR*) at Xq12 were also associated with POI (45). Indeed, *AR* is expressed in developing ovarian follicles, and *Ar* mutant female mice manifested a POI-like phenotype (2). Other X chromosomal genes may also be involved in POI. In this context, Castronovo et al. proposed that copy-number alterations in some autosomal loci, in combination with those in X chromosomal loci, may contribute to ovarian dysfunction (33).

## 4 Future perspectives

Impaired fertility is a major concern of women with Turner syndrome. Currently, several attempts including cryopreservation of ovarian tissues of prepubertal girls are made to preserve fertility of these women (11). Fleischer et al. reported that ovarian tissue cryopreservation was helpful for fertility preservation in women with Turner syndrome who have favorable predictive parameters such as 46,XX cell lines and spontaneous puberty (46). Further studies using ovarian tissue samples will serve to improve the reproductive outcomes in women with Turner syndrome.

## Author contributions

The author confirms being the sole contributor of this work and has approved it for publication.
